# Mapping and Exome Sequencing Identifies a Mutation in the *IARS* Gene as the Cause of Hereditary Perinatal Weak Calf Syndrome

**DOI:** 10.1371/journal.pone.0064036

**Published:** 2013-05-21

**Authors:** Takashi Hirano, Naohiko Kobayashi, Tamako Matsuhashi, Daisaku Watanabe, Toshio Watanabe, Akiko Takasuga, Mayumi Sugimoto, Yoshikazu Sugimoto

**Affiliations:** 1 Shirakawa Institute of Animal Genetics, Fukushima, Japan; 2 Laboratory of Animal Physiology, Faculty of Agriculture, Tokyo University of Agriculture, Kanagawa, Japan; 3 Hida Beef Cattle Research Department, Gifu Prefectural Livestock Research Institute, Gifu, Japan; 4 School of Veterinary Medicine and Animal Science, Kitasato University, Aomori, Japan; 5 National Livestock Breeding Center, Fukushima, Japan; University of Sydney, United States of America

## Abstract

We identified an *IARS* (*isoleucyl-tRNA synthetase*) c.235G>C (p.Val79Leu) substitution as the causative mutation for neonatal weakness with intrauterine growth retardation (perinatal weak calf syndrome). In Japanese Black cattle, the syndrome was frequently found in calves sired by Bull A. Hence, we employed homozygosity mapping and linkage analysis. In order to identify the perinatal weak calf syndrome locus in a 4.04-Mb region of BTA 8, we analysed a paternal half-sibling family with a BovineSNP50 BeadChip and microsatellites. In this critical region, we performed exome sequencing to identify a causative mutation. Three variants were detected as possible candidates for causative mutations that were predicted to disrupt the protein function, including a G>C (p.Val79Leu) mutation in *IARS* c.235. The *IARS* c.235G>C mutation was not a homozygous risk allele in the 36 healthy offspring of Bull A. Moreover, the IARS Val79 residue and its flanking regions were evolutionarily and highly conserved. The IARS mutant (Leu79) had decreased aminoacylation activity. Additionally, the homozygous mutation was not found in any of 1526 healthy cattle. Therefore, we concluded that the *IARS* c.235G>C mutation was the cause of hereditary perinatal weak calf syndrome.

## Introduction

The incidence of perinatal mortality in Japanese Black cattle is 4.5%, with 27.7% of the cases caused by neonatal weakness, and without any apparent clinical symptoms. In total, 72% of neonatal deaths caused by neonatal weakness are associated with normal gestation periods and low birth weights. This suggests intrauterine growth retardation; also known as perinatal weak calf syndrome [1]. Moreover, calves with this syndrome exhibit anaemia, depression, weakness, variable body temperature, astasia, difficulty nursing, growth retardation, and increased susceptibility to infection [2]. The pathological features of perinatal weak calf syndrome are anaemia with bone marrow dysfunction and foeto-placental dysfunction. The incidence of perinatal weak calf syndrome is dependent on the paternal and maternal family, and genetic factors have been implicated [1]. However, no genetic factors have been identified as the cause of perinatal weak calf syndrome.

Of the 538,111 Japanese Black calves born in 2010, the number of calves that lived less than 3 months was 22,020 (4.1%); in Holstein calves, the rate was 6.3% (34,182/538,656), according to a Japanese bovine individual identification database administered by the National Livestock Breeding Center (https://www.id.nlbc.go.jp/html/oshirase_back.html#2012012001). In offspring sired by Bull A, we found that the frequency of calf mortality before the age of 3 months was high (8.4%; 45/526) and diagnosed them with perinatal weak calf syndrome. Bull A was not a carrier of any known genetic diseases in Japanese Black cattle (e.g., BAND3 deficiency, factor XIII deficiency, claudin-16 deficiency, molybdopterin cofactor sulfurase deficiency, Chediak-Higashi syndrome, factor XI deficiency, and Marfan syndrome-like disease) [3]–[9]. Thus, unknown genetic factors were implicated in the high frequency of death in calves sired by Bull A.

Next-generation sequencing technology has enabled researchers to identify variants in individuals by whole-genome resequencing [10]. Recently, DNA sequence capture techniques have made it possible to determine sequences of exons and their flanking regions by whole-exome sequencing. The Human Gene Mutation Database (http://www.hgmd.org) shows that 95.1% of mutations are missense/nonsense mutations, splice site mutations, and small indels in coding regions of nuclear genes that underlie or are associated with inherited disease. In humans, protein-coding sequences constitute less than 2% of the whole genome. Whole-exome sequencing has been used to analyze human genetic diseases because it is efficient and cost-effective [11]–[14]. Therefore, we used whole-exome sequencing, which identifies variants from exons and their flanking regions, to identify the causative mutation for perinatal weak calf syndrome. To perform whole-exome sequencing, we prepared a bovine whole-exon capture custom array (Nimblegen) that targeted 174,377 exons. This approach enabled us to identify the causative mutation responsible for the hereditary perinatal weak calf syndrome.

To efficiently remove genetic defects from livestock populations such as cattle, it is important to establish a mating control based on the results of DNA tests that detect disease-caused mutations. We used mapping and exome sequencing to identify the mutation responsible for perinatal weak calf syndrome.

## Results and Discussion

### A Critical Region Detected in BTA 8

**Figure 1:**
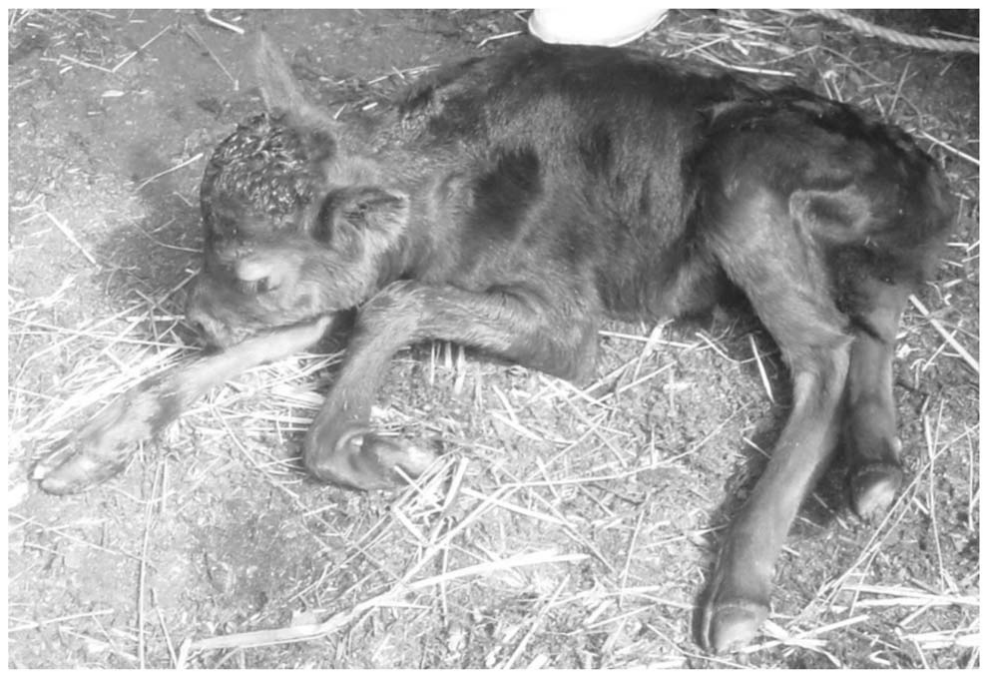
A calf with perinatal weak calf syndrome. The birth weight was 16 kg (normal average; 28.4±4.24 kg). The gestation period was 296 days (normal average; 288.9 days). The affected calf was weak and had difficulty nursing. The calf died at 2 days old.

To identify the critical region for perinatal weak calf syndrome, we constructed a paternal half-sibling family of offspring sired by Bull A. Fourteen calves with birth weights less than 20 kg despite normal gestation periods, weakness, and difficulty nursing were selected as the affected animals from among the calves that died before the age of 3 months (Fig. 1). A great-grandsire of Bull A was found in the fourth generation of the maternal ancestors of these calves, which suggested that perinatal weak calf syndrome was a recessive disorder.

**Figure 2:**
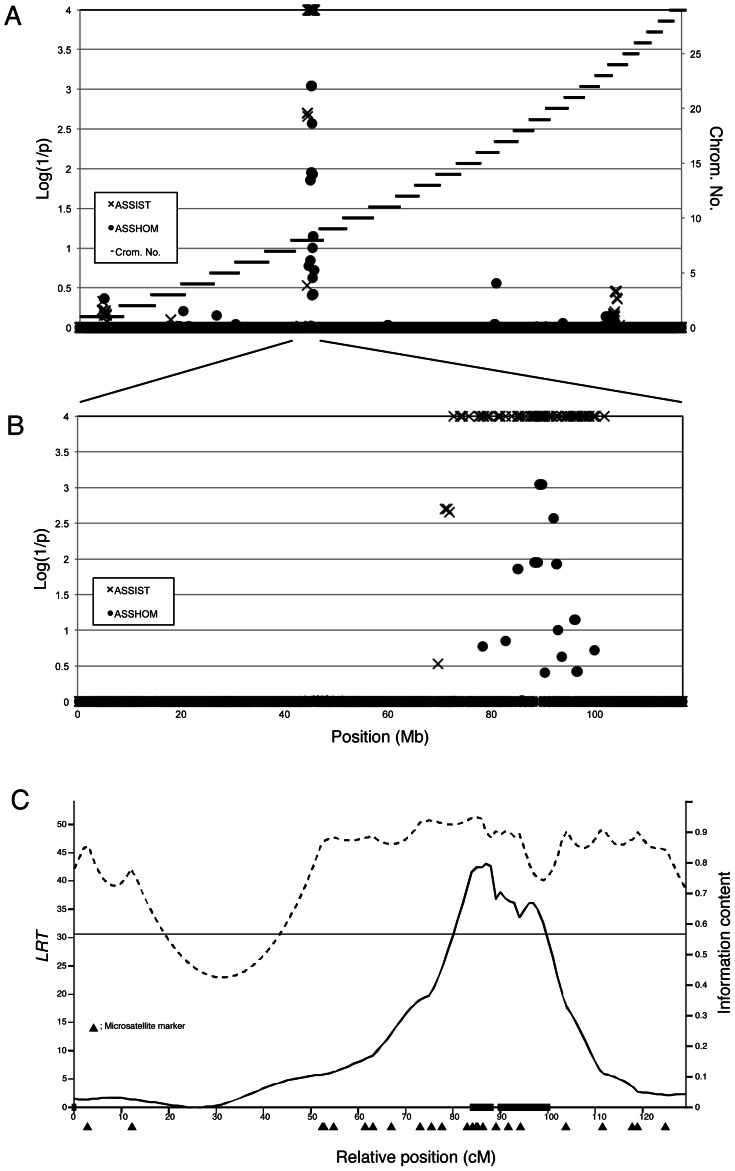
Genome-wide homozygosity mapping of perinatal weak calf syndrome. (A) The results of the genome-wide homozygosity mapping of perinatal weak calf syndrome with a paternal half-sib family composed of 13 affected and 30 normal animals sired from Bull A using ASSHOM (•) and ASSIST (X). The black horizontal bars mark the limits between the 29 autosomes. (B) The results of the homozygosity mapping on BTA 8. (C) The *LRT*-statistic profile for the disease on BTA 8. The horizontal line indicates the threshold for the 1% chromosome-wise significance level. The dashed line indicates information content (right *y*-axis). Microsatellite positions are indicated as filled triangles under the *x*-axis, respectively. The filled boxes on the *x*-axis represent the 95% confidence interval. The 95% confidence interval was flanked by *IDVGA-52* (79.3 Mb) and *MNB-38* (102.6 Mb), corresponding to the results of homozygosity mapping.

**Figure 3:**
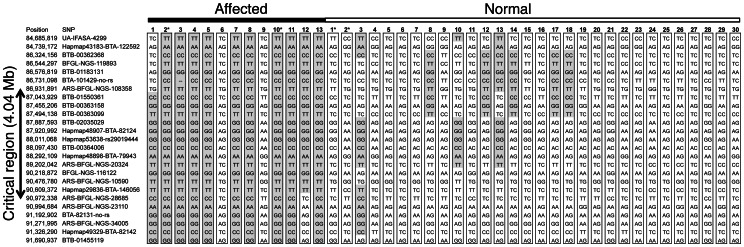
Genotypes of affected and normal animals for SNPs on BTA8. Overlapping blocks of extended homozygosity in the affected animals are shown in grey. The interval flanked by *ARS-BFGL-NGS-108358* and *ARS-BFGL-NGS-28685* (4.04 Mb) with shared homozygosity in 11/13 affected animals is determined as the critical region. In normal animals, grey blocks indicate the same homozygous haplotype shown in the affected animals with at least 6 SNPs.

To perform homozygosity mapping, we utilized the paternal half-sibling family. This comprised of 13 affected calves and 30 normal cattle sired by Bull A. The paternal half-sibling family was genotyped with the BovineSNP50 BeadChip. Homozygosity mapping was performed with 13,208 SNPs located in autosomes that were heterozygous in Bull A. The specific homozygous region in the affected offspring was detected on the distal half of BTA 8, at around 90 Mb (Figs. 2A and 2B). Furthermore, we performed a linkage analysis with 14 affected calves, 36 normal cattle, and 24 informative microsatellite markers covering BTA 8. The same region was repeatedly detected as the causative region (1% chromosome-wise significance level). The 95% confidence interval was calculated as the region from 82.9 cM to 103.6 cM flanked by *IDVGA-52* and *MNB-38* (20.7-cM interval) (Fig. 2C). The *IDVGA-52* and *MNB-38* positions in btau4.0 were 79.3 Mb and 102.6 Mb, indicating that the interval corresponded to the homozygosity mapping results. Based on the BovineSNP50 BeadChip genotyping, 11 of 13 affected calves (84.6%) shared the homozygous haplotype from 87,043,929 bp to 90,609,372 bp flanked by *ARS-BFGL-NGS-108358* and *ARS-BFGL-NGS-28685* on BTA 8. Therefore, we determined that the region flanked by these SNPs (4.04 Mb) was the critical region (Fig. 3). These findings indicated that the syndrome found in calves sired by Bull A was an autosomal recessive disorder. The mapping results provided the first evidence of a defective phenotype in perinatal weak calf syndrome that was clearly classified as an inherited disorder. This suggests that the phenotype of the remaining 2 calves among the 13 affected calves was caused by different pathogenic factors.

### Exome Sequencing Detects Causative Mutation Candidates

The critical region spanned 4.04 Mb and encompassed 29 annotated genes. To identify a causative mutation from these genes, we performed exome sequencing with 2 risk-haplotype homozygous affected calves (Affected-2, 10), 1 risk-haplotype heterozygous normal animal (Normal-1), and 1 no risk-haplotype normal animal (Normal-2) (Fig. 3). Each sample was applied to 2 lanes of a flow cell, and 1 paired-end 40-bases read length run was performed. The yield bases for each sample were Affected-2, 4,666.7 Mb (110.6×); Affected-10, 4,895.1 Mb (116.0×); Normal-1, 4,894.4 Mb (116.0×); and Normal-2, 5,049.3 Mb (119.7×). Potential sequence changes including single-nucleotide variations (SNVs) and small insertions or deletions (indels) were discovered with these data reads (Affected-2; 194 SNVs and 18 indels, Affected-10; 195 SNVs and 14 indels, Normal-1; 262 SNVs and 13 indels, and Normal-2; 222 SNVs and 12 indels). To distinguish potentially pathogenic mutations from these SNVs and indels, we first selected some variants that were detected as homozygous in 1 or 2 of the affected calves and undetected in both normal animals. Then non-synonymous mutations, predicted as damaging with Polyphen-2 or SIFT, were selected. Indels causing frame shifts were not detected. This occurred because it was suggested that the point mutations at position +5 of the 5′ splice site were particularly prone to aberrant splicing [15]. Intronic variants located within 6 bases of the exon-intron junction were also selected. Under these criteria, 3 SNVs were detected. Sanger sequencing confirmed that these SNVs produced the expected genotype for Bull A and 4 animals used for exome sequencing (Table 1). These SNVs, *IARS* c.235G>C (p.Val79Leu) (NM_001101069.1), *LOC786526* c.3449A>G (p.Gln1150Arg) (XM_001254184.3), and *CENPP* c.663+3A>G (NM_001105615.1), were used for further analysis.

**Table 1:** Confirmation of 3 SNPs in *CENPP*, *IARS* and *LOC786526.*

	Bull A	Affected-2	Affected-10	Normal-1	Normal-2
MS haplotype	R/nonR	R/R	R/R	R/nonR	nonR/nonR
*CENPP* c.663+3A>G	A/G	G/G	G/G	A/G	A/A
*IARS* c.235G>C	G/C	C/C	C/C	G/C	G/G
*LOC786526* c.3449A>G	A/G	G/G	G/G	A/G	A/A

R; risk haplotype.

nonR; non-risk haplotype.

### Identification of the Causative Mutation

Considering the perinatal weak calf syndrome found in calves sired by Bull A was an autosomal recessive disorder, the causative mutation had to be homozygous for a risk allele in the affected calves. To determine a possible causative mutation from the 3 SNVs, 14 affected and 36 normal cattle used in the mapping were genotyped for 3 candidate causative SNVs. Only the *IARS* c.235G>C (p.Val79Leu) substitution was not detected as homozygous risk-allele “*C*” in normal animals (Table 2) and remained as a possible causative mutation. To determine whether the *IARS* c.235G>C substitution was closely linked to a causative mutation that could not be detected by exome sequencing, we sequenced all 33 exons of *IARS* with Sanger sequencing. Two novel heterozygous, non-synonymous SNVs were detected in Bull A: c.2435A>G (p.Glu812Gly) and c.3394C>T (p.Leu1132Phe). These amino acid substitutions were predicted as not damaging by PolyPhen-2 and SIFT. Furthermore, risk-allele homozygous animals for the two SNVs were identified from the 36 normal cattle (Table 2). Therefore, we determined that c.2435A>G and c.3394C>T SNVs were not the causative mutation.

**Table 2:** Genotyping with the Bull A family used for mapping.

	Affected			Normal			
	R/R	R/nonR	nonR/nonR	R/R	R/nonR	nonR/nonR	Total
*CENPP* c.663+3A>G	12	1	1	3	27	4	48
*IARS* c.235G>C	11	1	1	0	21	15	49
*LOC786526* c.3449A>G	12	1	1	6	22	7	49
*IARS* c.2435A>G	12	1	1	6	25	3	49
*IARS* c.3394C>T	12	1	1	8	23	3	49

R; risk allele.

nonR; non-risk allele.

**Figure 4:**
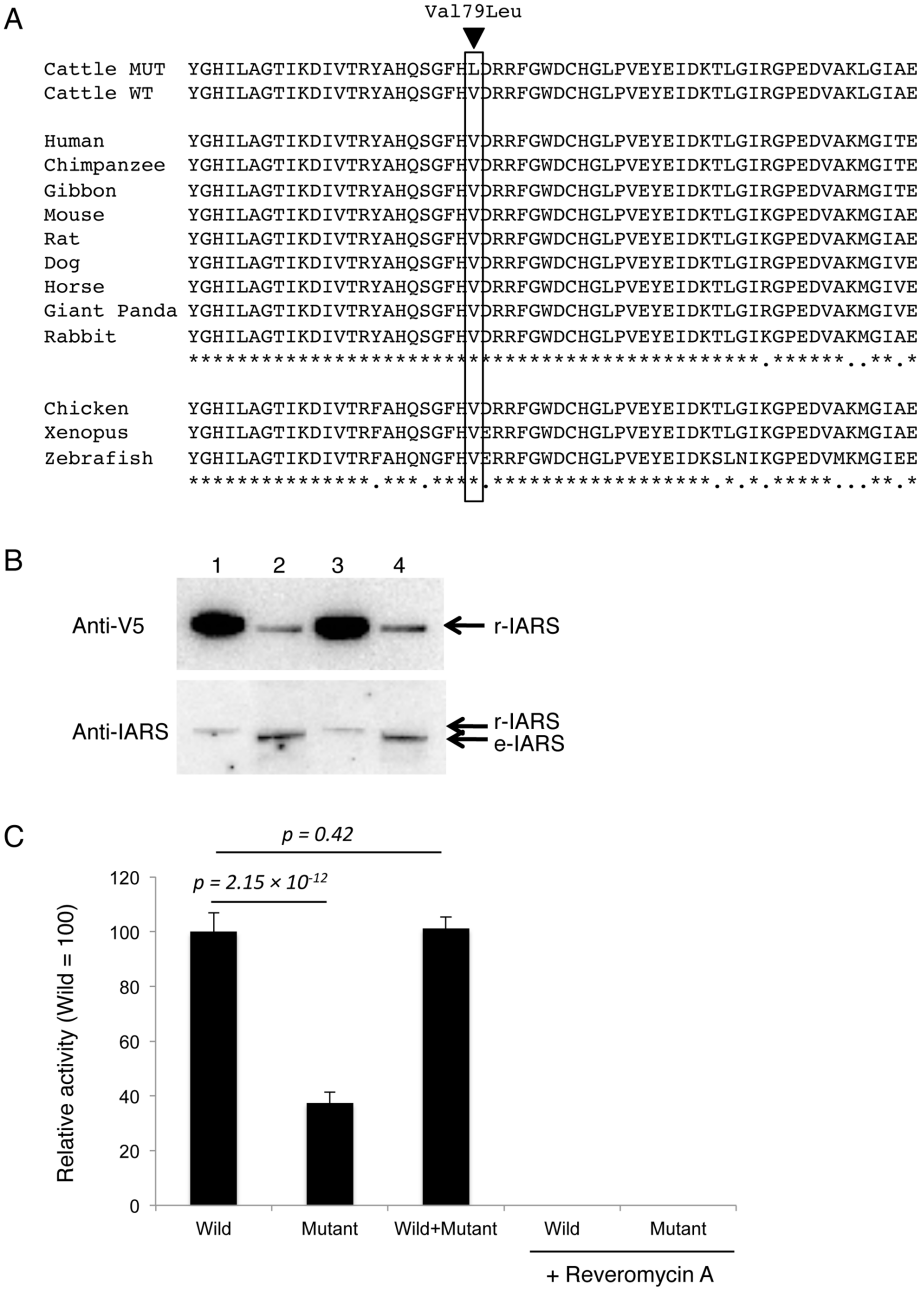
Alignment of the region flanking Val79Leu, and IARS aminoacylation activity. (A) Val79 is extremely conserved, and the flanking regions are also conserved in mammals. Top: cattle mutant and wild-type sequences. Middle: other mammalian sequences. Below: non-mammalian sequences. (B) NIH 3T3 cells were transfected with wild-type (Val-79) and mutant (Leu-79) V5-His-*IARS* cDNA plasmids. Whole-cell lysates were treated with His-tagged protein-binding magnetic beads (Dynabeads TALON). Immunoblot analysis was performed with anti-V5 antibody (Invitrogen) and anti-IARS antibody (H-219, Santa Cruz). 1. Purified wild-type V5-His-IARS. 2. Wild-type V5-His-*IARS*-transfected whole-cell lysate treated with TALON beads. 3. Purified mutant V5-His-IARS. 4. Mutant V5-His-*IARS*-transfected whole-cell lysate treated with TALON beads. Most of the V5-His-tagged protein was absorbed by TALON beads. The molecular weights of recombinant V5-His-IARS proteins (r-IARS) were higher than the endogenous murine IARS protein (e-IARS). The r-IARS fractions did not contain detectable e-IARS. (C) The aminoacylation activity of purified r-IARS (n = 3, in triplicate) shown with the standard deviation; 100% corresponds to 1.22 pmol/µg-protein, 30 min. Revelomycin A, 1 µg/ml. A *t*-test was conducted to obtain the *p* values.

The bovine *IARS* gene (*isoleucyl-tRNA synthetase*) encodes a protein containing 1,262 amino acids. Val79 is highly conserved, and the region flanking Val79 is also highly conserved in mammals (Fig. 4A). Val79 is located in a catalytic core domain of IARS (The Conserved Domain Database, NCBI). To determine the effect of p.Val79Leu mutation on IARS aminoacylation activity, we purified recombinant wild-type and mutant IARS proteins tagged with V5 and His (Fig. 4B). The aminoacylation activity of the mutant IARS protein was decreased to 38.0% (*p* = 2.15×10^−12^) (Fig. 4C). A mixture of equal amounts of wild-type and mutant IARS proteins demonstrated the expected combined activity (*p* = 0.42), suggesting that the mutant protein was not dominant-negative. Reveromycin A (1 µg/ml), an IARS-specific inhibitor [16], completely inhibited the aminoacylation activity of the wild-type and mutant IARS. The decreased aminoacylation activity caused by the p.Val79Leu mutation may have contributed to the incidence of perinatal weak calf syndrome.

Moreover, there were no risk-allele homozygous *CC* animals among the 1,526 normal cattle (146 offspring sired by Bull A and 1,380 healthy cattle unrelated to Bull A) (Table 3). In the normal population unrelated to Bull A, 194 of 1,380 (14.06%) animals were heterozygous. In this population, the frequency of the risk allele *C* was 7%, and it was expected that 7 animals (0.49%) among the normal cattle population would be the risk allele homozygous. However, homozygous *CC* animals were not detected in normal cattle. Moreover, a χ^2^ test revealed that the genotypic frequencies deviated from the Hardy-Weinberg equilibrium (*p*<0.01). These findings indicated that the risk allele homozygous *CC* genotype was specifically detected in the affected calves and suggested that these homozygous calves died or were culled because they did not exhibit normal growth. Thus, we concluded that *IARS* c.235G>C (p.Val79Leu) is the causative mutation for hereditary perinatal weak calf syndrome in the Bull A family.

**Table 3:** Further genotyping of *IARS* mutations.

	Sired by Bull A		Unrelated
c.235G>C	Affected	Normal	Normal
G/G	1	71	1,186
G/C	1	75	194
C/C	13	0	0
Total	15	146	1,380
C Freq.	0.9	0.26	0.07

The homozygous *CC* mutants might die during pregnancy. The *FANCI* deletion compromised fertility in Holstein-Friesian cattle; pregnancy failure was illustrated as the dams returned to estrus 56–270 days after insemination [17]. Artificial insemination (AI) is performed on dams in estrus, and the AI interval can appear as the interval from insemination to estrus. To determine whether the *IARS* mutation contributed to the death of an embryo or a fetus during pregnancy, we classified 2,597 AI intervals (60–365 days) according to the genotypes of the bull and dam. The frequency of AI intervals of 60–210 days was 66 of 311 (21.22%) in crosses between carrier bull × carrier dam, and 369 of 2,286 (16.14%) for others crosses, between carrier bull × normal dam, normal bull × carrier dam and normal bull × normal dam. The frequency of a 60- to 210-day AI interval in carrier bull × carrier dam was significantly higher (*p*<0.05) than the frequency in other crosses. Therefore, the *IARS* c.235G>C mutation may also have contributed to the death of an embryo or a fetus during pregnancy.

### DNA Test for Detecting the c.235G>C Mutation by Using the PCR-RFLP Method

The *IARS* mutant allele C frequency was 7% in the normal population unrelated to Bull A. A variant with >5% minor allele frequency is classified as a common variant [18]; thus it appears that the mutant allele commonly exists in Japanese Black cattle. To efficiently remove the mutation from Japanese Black cattle, a DNA-based test is needed. The c.235G>C mutation disrupted the *Hinc*II site, preventing the mutant allele sequence from being digested by *Hinc*II. Therefore, we developed a DNA-based test for detecting the mutation by using polymerase chain reaction-restriction fragment length polymorphism (PCR-RFLP) (Fig. S1).

Pinna tissues were collected from 200 calves that died before the age of 3 months, without any criteria for birth weight, weakness or difficulty nursing. These samples were genotyped for the c.235G>C mutation, and 41 of 200 (20.5%) animals were homozygous *CC* (Table 4). Therefore, it is expected that the DNA test may decrease the number of calf mortality before the age of 3 months by 20.5%. Moreover, 19 homozygous *CC* animals (34.5%) were detected in a population of 55 cattle with growth-retardation sired by heterozygous *GC* Bull B (Table 4). The sire of Bull B was the great-grandsire of Bull A. It is also possible that the DNA test may be useful for preventing the incidence of growth retardation.

**Table 4:** *IARS* mutations in random dead calves and growth-retarded calves.

	Dead calves	Growth reterdation
c.235G>C	before 3 months olda)	Sired by Bull Bb)
G/G	122	26
G/C	37	10
C/C	41	19
Total	200	55
C Freq.	0.30	0.44

a) Randomly collected without criteria for birth weight, weakness or difficulty nursing.

b) Bull B was heterozygous for the *IARS* mutation.

Aminoacyl-tRNA synthetases (ARSs) are ubiquitously expressed enzymes that are essential for the first step of protein synthesis. The aetiology of a number of diseases is connected to specific ARSs [19]. Mutations in *GARS*, *YARS, AARS* and *KARS* cause Charcot-Marie-Tooth (CMT) disease [20]–[23], and mutations in *DARS2* are responsible for leukoencephalopathy with brain stem and spinal cord involvement and lactate elevation (LBSL) [24]. The *SARS2* mutation was identified as a pathogenic cause of hyperuricaemia, pulmonary hypertension, renal failure in infancy, and alkalosis (HUPRA syndrome) [25]. A reduction of the aminoacylation activity of these mutant ARS proteins was shown and predicted. However, some GARS mutant proteins had full aminoacylation activity, this suggested that aminoacylation was not a fundamental cause of CMT disease [26]. The functional versatility of ARSs is being considered as a possible pathogenesis mechanism, but the role of tRNA synthetases in disease pathogenesis remains unclear [19]. Although the decrease in aminoacylation activity was detected in IARS mutants, a disruption of other possible IARS functions may contribute to the occurrence of hereditary perinatal weak calf syndrome.

## Materials and Methods

### Ethics Statement

Blood, semen or tissue samples were collected from bulls, dams, cattle, and calves by trained veterinarians following standard procedures and relevant national guidelines. The animal owners agreed that the samples could be used for our study. All animal experimentation was undertaken with the approval of the Commission of Shirakawa Institute of Animal Genetics (H20 - 8).

### Animal Samples

Bull A, 14 perinatal weak calf syndrome-affected calves and 146 normal cattle sired by Bull A, Bull B, 55 growth-retarded cattle sired by Bull B, 200 calves that died before the age of 3 months and 1,380 normal cattle unrelated to Bull A were used for the analysis. Calves with a birth weight less than 20 kg (normal average birth weight; 28.4±4.24 kg) despite a normal gestation period; weakness; difficulty nursing and a lifespan of less than 3 months were selected as affected. Healthy, normal-sized (carcass weight; >300 kg) that were collected in the slaughterhouse (Metropolitan Central Wholesale Market, Tokyo) were considered to be normal cattle. Cattle with a birth weight of 15–26 kg and a normal appetite were considered to have growth retardation cattle. Two hundred calves that died before the age of 3 months were included without criteria for birthweight, weakness or nursing. Genomic DNA was extracted from pinna tissues of affected calves, blood or adipose tissues of normal cattle, and the blood or semen of bulls according to standard protocols. The DNA concentration was adjusted to 100 ng/µl for the BovineSNP50 BeadChip assay and to 20 ng/µl for the microsatellite marker genotyping.

### Genotyping

Whole-genome genotyping was performed with BovineSNP50 BeadChip (Illumina). For genotyping with BovineSNP50 BeadChip, the integrity and fragmentation status of genomic DNA was verified with agarose gel electrophoresis, and 400 ng of DNA was used for the assay.

PCR conditions were optimized, as previously described [27], [28], for genotyping 24 microsatellite markers covering BTA 8, and Bull A was determined to be heterozygous at these markers. Genotyping was performed by using PCR with a fluorescence-labelled reverse primer, followed by electrophoresis with an ABI 3730 DNA Analyzer, as previously described [29]. These were analyzed with GeneMapper software (Applied Biosystems, Foster City, CA).

### Mapping

Homozygosity mapping was performed with the ASSIST and ASSHOM programs [30].

The sires' haplotypes were reconstructed by using the interval mapping method for half-sibling families [31], [32]. To test the hypothesis that a chromosomal location was related to the disease status, we used the following logistic regression model [33]:
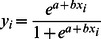
where *y_i_* is the disease status (0 or 1) and *x_i_* is the probability that the *i*th offspring inherited the sire’s first haplotype at a given chromosomal location. Parameters *a* and *b* were estimated at each location. The log likelihood of the alternative hypothesis and null hypothesis that *b* = 0 are



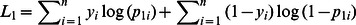
and



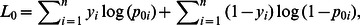
respectively, where 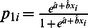 and 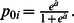


The test statistic 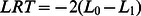. To determine the thresholds of the *LRT* statistics for chromosome-wise and experiment-wise significance, 10,000 random permutations of the phenotypic data were performed [34]. The disease locus location with 95% confidence intervals was estimated by the bootstrap method [35].

### Exome Sequencing

To perform bovine whole-exome sequencing, we prepared an exon sequence capture array (Nimblegen) covering 95% (42.2 Mb) of the 174,377 bovine exons.

Two risk haplotype homozygous syndrome-affected calves, one non-risk haplotype normal cattle and one risk haplotype heterozygous normal animal were selected based on microsatellite reconstructed haplotypes. DNA was extracted from pinna tissues of these animals for constructing sequencing libraries. Libraries for paired-end sequencing were prepared by using a paired-end sample prep kit (Illumina) following the manufacturer’s protocol. Five micrograms of DNA was fragmented by using a nebuliser, subjected to end repair, and ligated to adapters. The DNA fragments were separated by 2.0% agarose gel electrophoresis, and 300–350 bp fragments were eluted. One microgram of the constructed library was hybridized to the exon sequence capture array, and hybridized fragments were eluted as an exome sequencing library following the manufacturer’s protocol (Nimblegen). Each library was applied to 2 lanes of a flow cell, and paired-end sequencing was performed with a read length of 40 bases on a Genome Analyzer *IIx* (Illumina). Btau4.0 was used as the bovine reference genome. The reads were aligned to btau4.0 allowing two mismatches with GERALD.pl, and SNV and indel discovery was performed with run.pl in CASAVA software v1.7 (Illumina).

The effects of the detected missense mutations on protein function were analysed with PolyPhen-2 (http://genetics.bwh.harvard.edu/pph2/) and SIFT (http://sift.jcvi.org/) [36], [37].

### Sanger Sequencing

*IARS* c.235G>C (p.Val79Leu), *LOC786526* c.3449A>G (p.Gln1150Arg) and *CENPP* c.663+3A>G, detected by exome sequencing, were validated with direct sequencing using the following primers: *IARS*: F 5′-TTACCTTCTATGATGGGCCTC-3′ and R 5′-TTAACATCCCTGCCCTATGAC-3′; *LOC786526*: F 5′-GAGGCATAAACCAGGAGAGC-3′ and R 5′-TGATCTACTTTCGACTGGTCC-3′; *CENPP*: F 5′-AGATTTTCAGGGAATGCAAGAC-3′, and R 5′-ACATCTCAAGATGTCAGTTAGG-3′.

To sequence all *IARS* exons, primers flanking the exons were designed for each intronic region (Table S1). PCR was performed by using the genomic DNA of Bull A.

Amplified products were directly sequenced using the Big Dye terminator kit and the ABI 3730 DNA Analyzer (Applied Biosystems).

### IARS Aminoacylation Activity

Bovine wild-type and p.Val79Leu-mutated *IARS* cDNA sequences were prepared from bovine thymus cDNA using an In-fusion Advantage PCR cloning kit (Clontech). The primers used in the preparation are shown in Table S2. The prepared cDNAs were subcloned into pcDNA3.1/V5-His-TOPO (Invitrogen). The plasmid was transfected into mouse NIH 3T3 cells with Lipofectamine 2000 (Invitrogen). The transfected cells were lysed in cold phosphate-buffered saline (PBS) containing 1% Triton X-100 and a proteinase inhibitor cocktail (Sigma-Aldrich). V5-His-IARS protein was purified with Dynabeads TALON (Invitrogen). The eluted fraction was concentrated with an Amicon ultra-centrifugal filter device (Millipore) and diluted with PBS containing 0.1% Triton X-100. The protein concentration of the purified IARS was measured by protein assay (BioRad).

The aminoacylation activity of purified V5-His-IARS was determined as previously described [38]. The 20 µl reaction mixture contained 50 mM HEPES (pH 7.4), 20 mM KCl, 5 mM DTT, 5 mM MgCl_2_, 4 mM ATP, 1 mM spermine, 1 mg/ml yeast total tRNA (Invitrogen), 0.05 µCi L-[^14^C]isoleucine (7.5 µM:334 mCi/mmol), and purified IARS protein (1.0–1.3 µg). The omission of ATP was used as a blank. Reactions were incubated with the enzyme for 30 min at 30 °C. The rate was time dependent up to 30 min under the conditions. The reaction was terminated by adding 70 µl of 180 mM NaOAc/HOAc (pH 3.0) and 1 mg/ml salmon sperm DNA (Sigma-Aldrich). An equal volume of 20% trichloroacetic acid (TCA) was added to precipitate the nucleic acids. A reaction tube was briefly agitated by mixing and then centrifuged for 15 min at 15,000 rpm at 4°C. The precipitate was washed three times with 200 µl of 5% TCA containing 100 mM isoleucine, followed by washing once with 95% ethanol. The precipitate was solubilised with 70 µl of 100 mM NaOH. Radioactivity was quantified in a scintillation counter (Beckman Coulter).

### Artificial Insemination Interval Data

To analyze whether the *IARS* c.235G>C mutation contributed to the death of an embryo or fetus, data from 2,597 artificial insemination (AI) intervals of dams that received AI again from 60 to 365 days after the initial AI were collected. These AI intervals were classified as crosses between carrier bull × carrier dam or crosses of other pairs, according to the genotype of the bull and dam. The frequencies of AI intervals shorter than 210 days in each group were analyzed by χ^2^ test.

### IARS c.235G >C Detection with PCR-RFLP

Mutation detection by PCR-RFLP was performed by *Hinc*II digestion of the PCR products with the primers for *IARS* c.235 G>C described above. Following *Hinc*II digestion, the normal PCR products were detected in the 276-bp and 116-bp digested fragments, and the mutant products only were detected in the 392-bp uncut fragment.

## Supporting Information

Figure S1 **The DNA test using the PCR-RFLP method.** Normal and mutant alleles can be distinguished by *Hinc*II digestion of PCR products. The undigested fragment indicates the mutant allele, and digested fragments indicate the wild-type allele. Each genotype was determined by direct sequencing. The same genotypes were obtained by PCR-RFLP.
(TIF)

Table S1 **Primers used to sequence all exons of the bovine** ***IARS*** **.**
(PDF)

Table S2 **Primers used to prepare the wild-type and mutant** ***IARS*** **cDNA.**
(PDF)
