# Supplementary figures and images for: Mapping and Exome Sequencing Identifies a Mutation in the IARS Gene as the Cause of Hereditary Perinatal Weak Calf Syndrome
Source: PLoS One. 2013 May 21;8(5):e64036. doi: 10.1371/journal.pone.0064036 (PMC3660308; doi:10.1371/journal.pone.0064036)

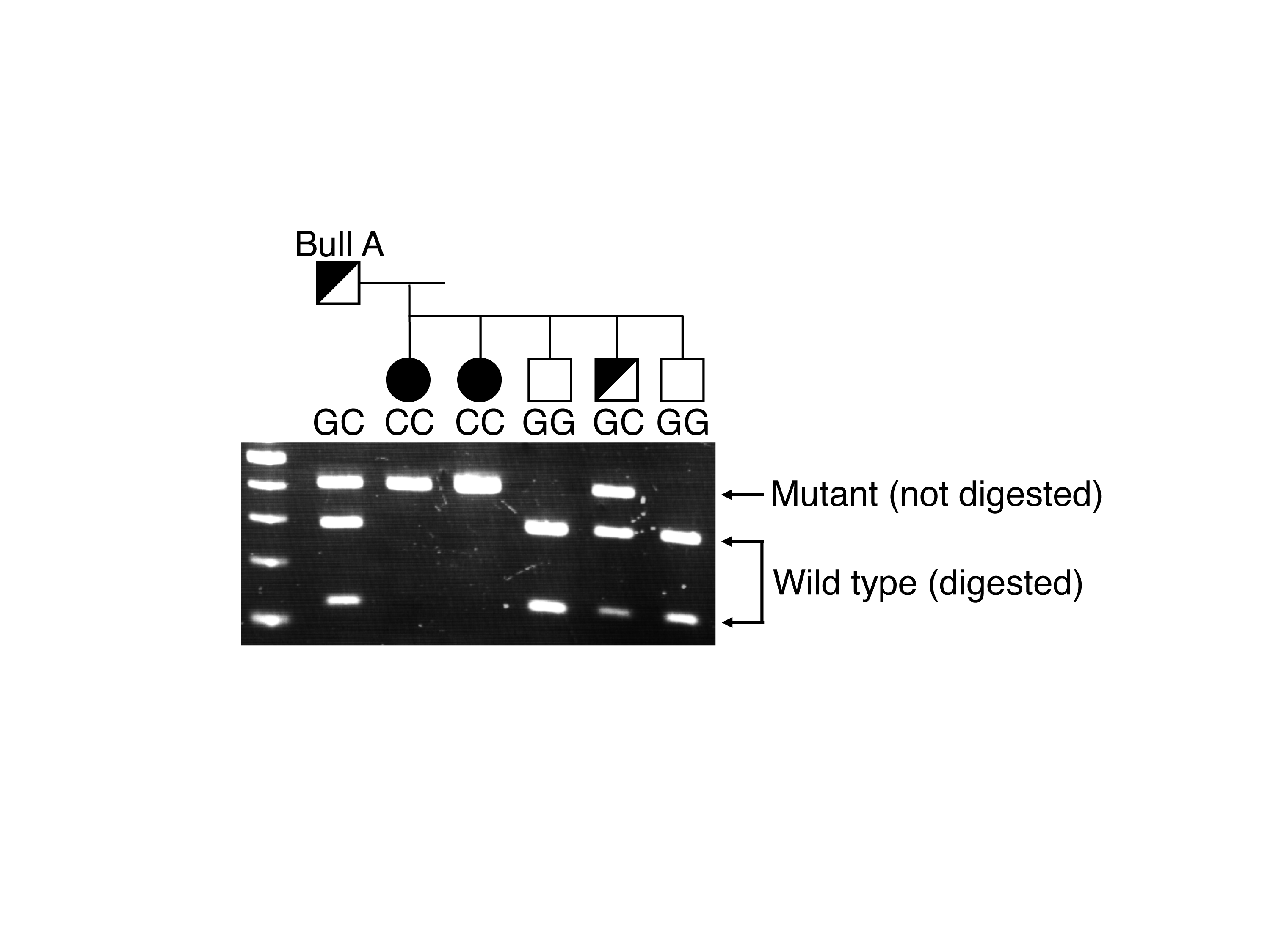

Supplement: Figure S1 — The DNA test using the PCR-RFLP method. Normal and mutant alleles can be distinguished by HincII digestion of PCR products. The undigested fragment indicates the mutant allele, and digested fragments indicate the wild-type allele. Each genotype was determined by direct sequencing. The same genotypes were obtained by PCR-RFLP. (TIF) [file pone.0064036.s001.tif]
