# Supplementary material for: Mapping and Exome Sequencing Identifies a Mutation in the IARS Gene as the Cause of Hereditary Perinatal Weak Calf Syndrome
Source: PLoS One. 2013 May 21;8(5):e64036. doi: 10.1371/journal.pone.0064036 (PMC3660308; doi:10.1371/journal.pone.0064036)
Supplement: Table S1 — Primers used to sequence all exons of the bovine IARS . (PDF) [file pone.0064036.s002.pdf]

Table S1. Primer sequences for all exons of bovine *IARS*

	Primer sequence (Forward)	Primer sequence (Reverse)	Size (bp)
IARS exon 1	TACTGCAAGCTTCCCAGTGC	TCTTGTGAACCAGAGGGGAC	371
IARS exon 2	CCATATAGTATCATGGCTAGTG	GTGGAAAGGAAGAGACTTGAC	415
IARS exon 3	TGTCTGAGTTTCCTAGCCTTG	AGAGAAAATGAACTGTACATAC	440
IARS exon 4	GGTATGTTTGAGTCATCAGCC	GGACAGTCAACTTTGGGTGC	495
IARS exon 5	CAGTGTTGGACATTTGGTAGC	AGCTGATTCATACTAGAGCATC	341
IARS exon 6	TTTACCAGCTATTCACTGGTTG	CAAAACTAAGAGCACACGTGC	430
IARS exon 7	AGCTCAGCTTATTCTGAAAATC	AAAAAAGGAAGGAAATGTCAGC	343
IARS exon 8	TTGCCTAAGATTCCAGTCACG	CATTAGTGCTAGAACATGTGAC	382
IARS exon 9	TTCAAGTCACATGTTCTAGCAC	ATTTTTTTGGATTCAGGCACAC	314
IARS exon 10	CCCAATCTGAATTATTCTGTCC	ATCTCCATAGTCTGAACAACAG	361
IARS exon 11	GATTCTCTGAATGATCCTTGAG	CAAAACAAAACCAACTTCCTGG	420
IARS exon 12	AATAGCTGAGATATAGACCAAG	TTCCACACACTAACTTGAAAGG	373
IARS exon 13	CGTGGAGGGAGATGACATAG	CACGAGAGGGATTCTGCTTC	434
IARS exon 14	CATTCTCCTAGAAGTGAGTGC	AACCTGATGAAATAAAGGAACG	388
IARS exon 15	TTCCCACAGTCTTTCATGTGG	CTAGTGTTTGATCTCTTCATGG	333
IARS exon 16	TGTGAAAGCCTGTGATAATTGC	GGAGCAGAGCCTATCTTGTC	461
IARS exon 17	GATGATCCTGATATATCACCAG	AGAACAGGAACAGAACTGGTG	390
IARS exon 18	TGCCTTAATAGTAGCTTGTGTC	ATGATCAGCATCACCGAAACC	301
IARS exon 19	TTTGTCAGTTTTAAGCGGACTG	CATCTACGGCCTTTCCAACG	446
IARS exon 20	TTGGTTTGGCTAGTTCTTTCAG	TCTGAATCACTTTGCTGTACAG	387
IARS exon 21	GGAATGAAAACTGACCTATCTC	ATAGCTTCACCAAGTGAGAGC	354
IARS exon 22	CTTAATTATAGCTGAGTTTGCC	TACTCACAGATGATTACAGGAC	311
IARS exon 23	GTTGTATTAACCTCATGTCCAG	AGGAATAGCTCAACTGGAAGG	385
IARS exon 24	CACTATGTTCTCAAGAGCCTC	AGTCATTCACAGCCTTACTGC	377
IARS exon 25	TGCAGTAAGGCTGTGAATGAC	AACTGTAAGATTTCACAGATGG	347
IARS exon 26	TATCCTTTCATAGTCAATCAGC	TAAGGGAAGGCTGTAATAGTAG	409
IARS exon 27	TTTTGTGATTGAATGGTGCTGC	CTGAATGGATCGTAGATCAATG	359
IARS exon 28	TTTTCCCAGAGAATTTGTCAGG	TTCACTATTCTCCAGAGAAGAC	447
IARS exon 29	GGAAAGGTAGCTCTTGGCTG	TGTCTACTTCACTTCCTAGAAG	390
IARS exon 30	AAGACATGAATAGACATTGACC	GGCAGGAAGTACTGTAGAGG	383
IARS exon 31	GAAAGCGTCACTATAAAGGTAC	CAAAGAAACGTGGGCATCTAC	421
IARS exon 32	ATTTCTACAGTGAATCCCACTC	ATGGCAAATTTCTGGAGATAGC	453
IARS exon 33-1	AAGGTAAGGCTGAATATCTTGC	CTGAGGTAACACCAACTTTCC	436
IARS exon 33-2	TGAAGATGTTTCGTTCAGATGC	GGAAATAACTTGGTGCCAAATG	576
