# Supplementary material for: Mapping and Exome Sequencing Identifies a Mutation in the IARS Gene as the Cause of Hereditary Perinatal Weak Calf Syndrome
Source: PLoS One. 2013 May 21;8(5):e64036. doi: 10.1371/journal.pone.0064036 (PMC3660308; doi:10.1371/journal.pone.0064036)
Supplement: Table S2 — Primers used to prepare the wild-type and mutant IARS cDNA. (PDF) [file pone.0064036.s003.pdf]

Table S2 Primers used to prepare mutated IARS and wild type cDNA.

SeqName	Sequence	Size
IARSful1F	AAAATGGTTCAACAAGTTCCAGAAAACATCAGTTT	255
IARSful1wtR	GCCAAATCTTCTGTCAACGTGAAACCCACTCTGGT	
IARSful1mutR	GCCAAATCTTCTGTCAAGGTGAAACCCACTCTGGT	
IARSful2F	GACAGAAGATTTGGCTGGGATTGTCATGGTTTACC	530
IARSful2R	AGTAATTTTCCTCTGACAGAATCTTTAATCTTCAC	
IARSful3F	CAGAGGAAAATTACTCATTTTAATGGAAGCCAGAT	530
IARSful3R	TCAGCAGCTGATCCACCATGTGCTCCACCCTAACG	
IARSful4F	TGGATCAGCTGCTGAGGAACAATGACCTGTGTTAC	530
IARSful4R	TTGACCATCACTTGCCAGGACAAGTCCATTCACAA	
IARSful5F	GCAAGTGATGGTCAAAAAATGAGCAAACGCAAAAA	530
IARSful5R	GGGGCCATAAGTCTGCACAAAGACAGCAGAACACT	
IARSful6F	CAGACTTATGGCCCCCTATACCCCTTTTCTTACTG	530
IARSful6R	CTTCATGCAATTCATGGCCTTCCACAACAATGGTC	
IARSful7F	ATGAATTGCATGAAGAAGATATCCGTCTCATATAC	500
IARSful7R	TTTTGGATTTTCCAGAAGTAATACTCCACCTTGCT	
IARSful8F	CTGGAAAATCCAAAAGGAGATAATAGGTTGGACCT	
IARSful8R	GAAGTCTGCTGTAGTTGGTAACACAGAAACATACA	489
IARSful8SR	TTAGAAGTCTGCTGTAGTTGGTAACACAGAAACAT	492
